# Ophthalmia in the Army—A Contrast

**Published:** 1897-02-06

**Authors:** 


### Ophthalmia in the Army—A Contrast.

It is only by contrasting the present with the past that we become aware of the immense advances which have taken place in medical art and science in the course of the century. Nothing could show this better than the contrast drawn by Mr. John Tweedy, F.R.C.S., at the meeting of the Royal London Ophthalmic Hospital, between the manner in which ophthalmia, that Egyptian scourge, ran riot among the troops in Egypt at the beginning of the century, and the manner in which it was held in check by scientific measures in the case of our army which was landed there some years ago. He said that in the Egyptian campaigns at the beginning of this century, Napoleon landed in Egypt with 32,000 troops. Within two months, he believed that it was strictly true to say that every one of those men were attacked with ophthalmia, and thousands of them became blind. During the English campaign in Egypt at the commencement of this century similar disasters overtook the British soldiers. There was evidence with respect to one battalion of 700 men, that 636 were attacked with ophthalmia, and that 50 of them became totally blind in both eyes, 40 blind in one eye and that more or less damaged sight resulted to many others. Now what can we show by way of contrast as evidencing the advance we have made—the great good that the advancing knowledge of ophthalmic diseases has conferred upon individuals of the community and upon the nation itself. "Just at the time of the last Egyptian campaign of 1882," said Mr. Tweedy, "my friend Surgeon-General Marston, who was to have charge of the Sanitary Department, came to talk over with me what were the best means to be adopted to prevent ophthalmia, and, if it broke out among the soldiers, what were the best measures to save the sight. We agreed upon a certain plan, which, of course, had to be submitted to the authorities. When our soldiers went out to Egypt the most minute instructions were given to every soldier, to every officer, and to every medical officer in the army as to the conditions to be observed. The result of this was that all went well until the soldiers concentrated in Cairo after the battle of Tel-el-Kebir, when, perhaps, the precautions were relaxed. The consequence was that a fearful attack of ophthalmia broke out, and within a month quite one man in every ten was attacked. There were 1,800 severe cases of Egyptian ophthalmia. Thanks, however, to the observance of all that sanitary science could suggest, to the unflagging attentions of the army surgeons, and to the use of proper remedies, every one of those cases was cured, and no one man lost his sight." Surgeon-General Hunter, in reporting these circumstances, concludes with these words: "These results, when contrasted with those which have been recorded of the same disease in the wars of 1801, justify the expression of my opinion that the Army Medical Department has never performed a better service than this, or one that it has more reason to be proud of."

